# The Impact of Platelet-Rich Plasma Application during Cesarean Section on Wound Healing and Postoperative Pain: A Single-Blind Placebo-Controlled Intervention Study

**DOI:** 10.3390/medicina60040628

**Published:** 2024-04-13

**Authors:** Michał Barwijuk, Katarzyna Pankiewicz, Aleksander Gałaś, Filip Nowakowski, Patrycja Gumuła, Artur J. Jakimiuk, Tadeusz Issat

**Affiliations:** 1 Department of Obstetrics, Women’s Diseases and Oncogynecology, National Institute of Medicine of the Ministry of Interior and Administration, Woloska 137, 02-507 Warsaw, Poland; barwijuk@gmail.com (M.B.); jakimiuk@yahoo.com (A.J.J.); 2 Department of Obstetrics and Gynecology, Institute of Mother and Child, Kasprzaka 17a, 01-211 Warsaw, Poland; patrycja.gumula@imid.med.pl (P.G.); tadeusz.issat@imid.med.pl (T.I.); 3 Chair of Epidemiology and Preventive Medicine, Department of Epidemiology, Jagiellonian University Medical College, 7 Kopernika St., 31034 Krakow, Poland; aleksander.galas@uj.edu.pl; 4 Center for Reproductive Health, Institute of Mother and Child, Kasprzaka 17a, 01-211 Warsaw, Poland

**Keywords:** platelet-rich plasma, cesarean section, wound healing, postoperative pain

## Abstract

*Background/Objectives*: The aim of this study was to evaluate if platelet-rich plasma (PRP) application into the wound during cesarean delivery improves wound healing and reduces pain in the postoperative period. *Materials and Methods*: A total of 46 patients undergoing cesarean section (CS) were included in this single-blind placebo-controlled intervention study: 23 women in the PRP group and 23 in the placebo group. Every patient was asked to evaluate pain by using the Visual Analogue Scale (VAS) immediately after surgery, as well as 6 and 12 h after the surgery. The use of analgetics was also recorded. The postoperative scar was assessed using the Patient and Observer Scar Assessment Scale (POSAS). *Results*: There was no case of wound dehiscence in either group. Significant differences between the groups in the scar quality assessment were detected in both patient and doctor POSAS results on days 8, 30 and 90 after surgery in the favor of the PRP group. There was no difference in the pain intensity assessment on the VAS recorded after surgery, but PRP patients required fewer paracetamol doses per day than the control group. *Conclusions*: PRP application during CS significantly improved wound healing in both short- and long-term assessment. Although it did not influence postoperative pain intensity, it may reduce the use of analgetics after surgery.

## 1. Introduction

Platelet-rich plasma (PRP) is an autologous plasma, rich in growth factors, a platelet-rich fibrin (PRF) matrix and platelets [1]. The term PRP was first introduced by hematologists in 1970 to describe plasma with an increased concentration of platelets compared with peripheral blood, intended for patients with thrombocytopenia [2]. PRP is currently one of the most commonly used preparations in regenerative medicine, because it contains a high concentration of growth factors and cytokines participating in various cellular, immune and regenerative processes, such as wound healing and tissue regeneration [3]. Specific growth factors and cytokines in PRP include, i.e., transforming growth factor-beta (TGF-β), fibroblast growth factor (FGF), platelet-derived growth factor (PDGF), insulin-like growth factors 1 and 2 (IGF-1 and IGF-2), vascular endothelial growth factor (VEGF) and epidermal growth factor (EGF) [3]. PRP therapies are widely used in orthopedics, dermatology, plastic surgery, cardiothoracic surgery, dentistry and diabetic wound healing [4,5,6,7]. There is also an increasing amount of research concerning its use in gynecology—in treatments for infertility, Asherman’s syndrome or premature ovarian insufficiency (POI)—as well as for urinary incontinence and other lower genital tract symptoms [8,9,10,11]. In reproductive medicine, PRP intrauterine infusion is used in patients with thin endometrium or Asherman’s syndrome to induce endometrial growth and increase clinical rates of pregnancies [8,11]. In women with POI or diminished ovarian reserve, PRP can be injected under ultrasound guidance into the ovarian cortex to increase anti-Mullerian hormone levels and decrease follicle-stimulating hormone (FSH) concentration, thus improving the reproductive outcomes (number of oocytes retrieved, clinical pregnancy and live birth rates) [8,9]. In urogynecology, PRP injections into the different pelvic ligaments may improve the symptoms of genital prolapse and urinary incontinence, being an important alternative for vaginal implants used in pelvic floor reconstruction procedures that are known to have numerous serious adverse effects [10,11]. Noteworthily, the equipment used to produce PRP and the injections themselves have been cleared by the FDA, but since PRP is a substance derived from the patient’s own blood, it is not considered a drug. FDA clearance means that doctors can prescribe and administer PRP if they believe it is in the best interest of the patient [12].

Cesarean section (CS) is one of the most practiced surgeries in the world. Recently, CS rates have rapidly increased worldwide, achieving a global rate of about 21% [13,14]. In some regions, however, this percentage differs significantly from the average, with the lowest in sub-Saharan Africa and the highest in Latin America [15]. Projections showed that by 2030, 28.5% of women worldwide will give birth by CS (38 million cesareans will be performed per year) [13,16]. Noteworthily, CS is related to a 2-fold increase in maternal morbidity in comparison to vaginal delivery, with infections (including surgical site infection) as the most common complications [17,18]. It is estimated that about 3 to 15% of patients undergoing CS suffer from wound complications, such as dehiscence, seroma, hematoma and superficial infections [19,20]. This leads to prolonged hospitalization, use of antibiotic therapy and increased postpartum costs, applying an incremental burden on the healthcare system. Simultaneously, evidence-based actions in the peri-operative management of patients submitted to surgery can beneficially modulate their recovery, especially when a patient needs to take care of a newborn child [14]. According to the FIGO good practice recommendations and enhanced recovery after surgery (ERAS) protocols, the current management strategies for wound healing after CS include prophylactic antibiotics within 60 min of CS and prior to skin incision, chlorhexidine–alcohol for skin antisepsis with 3 min of drying time prior to incision, closure of the subcutaneous layer if it is ≥2 cm in depth, and subcuticular skin closure with sutures rather than with staples [20,21]. The available evidence does not support a recommendation for any particular type of wound dressing, and some possible procedures are not recommended during CS, such as routine use of wound drains or rectus muscle reapproximation (because this increases postoperative pain without any benefit) [14,21]. The use of PRP has been evidenced to improve wound healing in different groups of patients; however, there are only a few studies concerning its use during CS. Tehranian et al. revealed that the use of PRP was associated with better wound healing in both short- and long-term assessments, as well as with a reduction in the intensity of postoperative pain [22]. The second study published by Elkhouly et al. cannot be taken into consideration because of the expressions of concern that have been published by Karger [23,24].

The aim of this study was to evaluate if PRP application into the wound during cesarean delivery improves wound healing and reduces pain in the postoperative period. This is one of the first studies concerning the use of PRP during CS in the context of wound healing and postoperative pain and the first study assessing not only the intensity of pain but also the use of analgetics. We hypothesized that PRP application during abdominal closure during CS may improve wound healing in both short- and long-term assessments and may also reduce postoperative pain and the use of analgetics.

## 2. Materials and Methods

### 2.1. Study Population

In this single-blind placebo-controlled intervention study, adult women (>18 years of age) with uncomplicated pregnancies undergoing elective CS in the Department of Obstetrics, Women’s Diseases and Oncogynecology, National Medical Institute of the Ministry of the Interior and Administration in Warsaw, Poland, between January 2018 and May 2019 were included. Patients were randomly assigned to one of the two different groups: group 1, patients who received the application of PRP into the wound during the surgery, and group 2 (control group), patients who received the application of a placebo (0.9% NaCl solution). The allocation ratio was 1:1, and the study was supervised by an independent clinician, who was not involved in the PRP application during surgery. The randomization was performed manually and the allocation concealment was performed by using sequentially numbered opaque envelopes. The study was single-blind, meaning the participants were unaware of the treatment they received.

The primary outcome measured was the wound dehiscence, whereas the secondary outcomes were postoperative pain intensity, the use of analgetics after surgery, scar quality assessment and quality of life assessment after surgery. The exclusion criteria included the following: 1. pregnancy complications, including gestational diabetes, hypertensive disorders of pregnancy, intrahepatic cholestasis, eclampsia/preeclampsia and coagulation disorders; 2. obesity (BMI > 40 kg/m^2^); 3. urgent CS; 4. allergy to analgetics; 5. viral or bacterial local infections. This study was approved by the Bioethical Committee of the Central Clinical Hospital of the Interior in Warsaw (approval code: 99/2016; approval date: 17 October 2016), and informed consent was obtained from all patients. A sample size of participants (23 treatment participants, 23 control participants) was estimated by power analysis to achieve greater than 80% power to detect a 35% change in the incidence of wound dehiscence (using 95% CI).

### 2.2. Surgery and Postoperative Treatment

Elective cesarean section was performed by using the Misgav-Ladach technique with abdominal opening by the Joel-Cohen method, as was described before [25,26]. At the end of the CS, during the abdominal closure, PRP or a placebo (0.9% NaCl solution) was applicated by a series of microinjections into the abdominal muscles’ fascia and the subcutaneous tissue. All patients had subdural anesthesia during the procedure. In the early postoperative period, all patients were treated with analgetics. The basic therapy was intravenous paracetamol and morphine given in the form of patient-controlled analgesia (PCA). Additionally, when needed, some patients were treated with intravenous ketoprofen. Patients were requested to evaluate the pain by using the Visual Analogue Scale (VAS) immediately after the surgery and then 6 and 12 h after the surgery. We used a VAS version with a horizontal line with pointed numbers from 0 to 10, where 0 was marked as “no pain” and 10 was marked as “the worse pain you can imagine”. Patients were asked to answer the following question: “How can you describe the intensity of your pain using presented numbers?” (see Supplementary Materials). The postoperative pain was assessed during rest. The use of analgetics was measured as the mean and total numbers of morphine boluses needed, as well as the number of doses of paracetamol, metamizole and ketoprofen needed per day.

### 2.3. Scar Quality Assessment

The Patient and Observer Scar Assessment Scale (POSAS) was used to evaluate the appearance of the postoperative scar. The POSAS is a reliable and valid scar assessment scale that measures scar quality from two perspectives: those of the patient and the clinician (the observer). It includes the assessment of 6 parameters for both the observer and patient. These are vascularity, pigmentation, thickness, relief, pliability and surface area. Each parameter can be scored on a 1-to-10 scale, where the lowest score of “1” indicates normal skin and the highest score of “10” indicates the worst scar imaginable (see Supplementary Materials) [27]. In the presented study, the POSAS result was recorded on days 1, 8, 30 and 90 after surgery by both the patient and the doctor.

### 2.4. Quality of Life Assessment

Quality of life after CS was assessed by the use of the SF-12 questionnaire, including 8 life domains: limitations in physical activities because of health problems, limitations in social activities because of physical or emotional problems, limitations in usual role activities because of physical health problems, bodily pain, general mental health (psychological distress and well-being), limitations in usual role activities because of emotional problems, vitality (energy and fatigue) and general health perceptions. All patients were asked to fill out the questionnaire on days 1, 8, 30 and 90 after surgery.

### 2.5. PRP Preparation

PRP was prepared using the closed method and the gel separation technique with a commercially available kit. The blood of the patient was collected by venipuncture in a sterile tube with a special gel–chemical polymer, specifically 1–2 mL of a thixotropic polymer, enabling very efficient separation of morphotic elements from plasma (Regeneris^®^, Regen Lab SA, Le Mont-sur-Lausanne, Switzerland, European directive certificate no. 93/42/EEC). This was followed by centrifugation for 5 min at 1500× *g* (single spin method). After centrifugation, PRP was present above the separating gel. The gel has a specific gravity lower than that of red blood cells and white blood cells, but higher than that of platelets. On centrifugation, the gel settles between the plasma containing platelets and all other components below. The final step was the addition of platelet-activating factor (thrombin). Then, the PRP was ready to use during the surgery. In this protocol, about 8–10 mL of PRP was derived from 24–30 mL of patients’ whole blood with platelet recovery of 90 ± 5%, and a platelet-derived growth factor ab (PDGFab) concentration of 140 ± 14 ng/mL was obtained.

### 2.6. Statistical Analysis

Statistical analysis was run using the intent-to-treat (ITT) approach. The basic characteristics of the study participants were presented by descriptive statistics using the mean with standard deviation for continuous variables and numbers with percentages for categorized variables. Next, as the investigated sample size was low, which is associated with low power while testing for normal distribution, the nonparametric U-Mann–Whitney test was used for continuous variables. The chi-squared test for categorical variables was run if the assumption of expected values being no more than 5 was met; otherwise, Fisher’s exact test was used. As there were repeated observations made on the 8th, 30th and 90th days, the repeated measures ANOVA was used to reveal the presence of difference in the POSAS scores. Finally, the growth mixture models with a linear prediction of dependent variable changes over time were run to answer the questions of whether the curves representing the change in the POSAS score over time differ by groups when the distances in time-point measures are considered. If the linear prediction was not observed, the quadratic prediction was tested as well (assuming that the nature of the change may be not clearly linear, but also like a quadratic function). A value of *p* < 0.05 was taken as the significance level in the above-mentioned analyses.

## 3. Results

**Figure 1:**
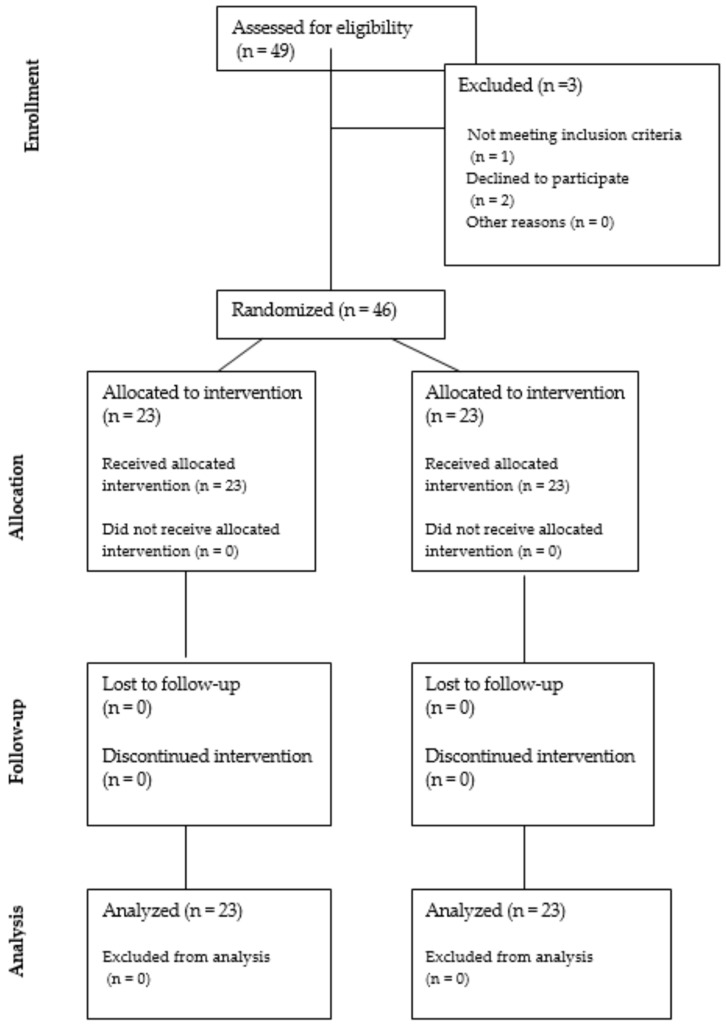
The CONSORT flowchart for patient recruitment and analysis.

A total of 46 patients were included in this study: 23 women in the PRP group and 23 in the placebo group. The CONSORT flowchart for patient recruitment and analysis is presented in Figure 1.

There was no difference between the groups in terms of age, BMI, parity or concomitant diseases. All women in this study were also non-smokers. The only difference between the groups was group B streptococcus colonization during pregnancy, which was significantly more common in the PRP group, applying to 10 (43.5%) vs. 3 (13%) patients (*p* = 0.022). The demographic and clinical characteristics of the study participants are summarized in Table 1.

The CS indications did not differ between the groups with the most common breech presentation and non-obstetric indications (i.e., ophthalmologic, orthopedic, cardiologic). There was no difference between the groups in terms of the gestational age at delivery, blood loss, length of hospitalization, neonatal birth weight or Apgar scores. Detailed delivery and neonatal outcomes are presented in Table 2.

**Figure 2:**
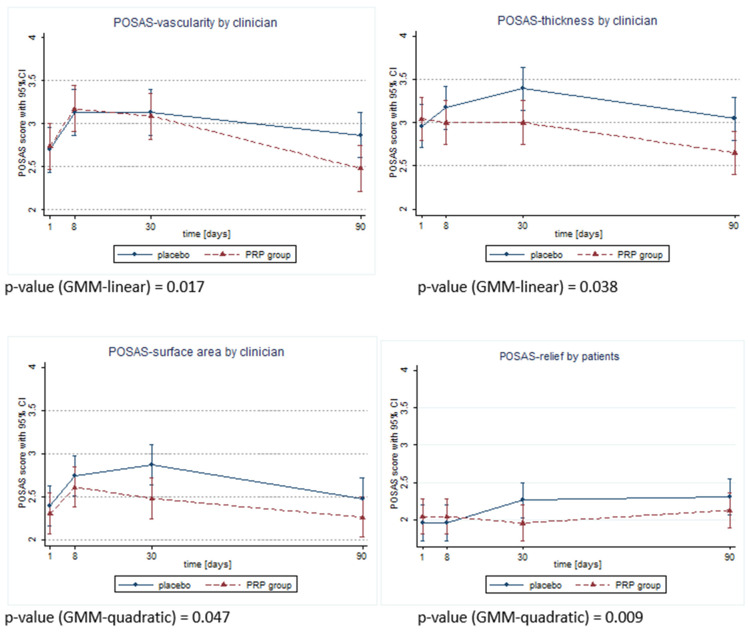
Differences in time trend changes across POSAS features observeId as significant by clinician or patient.

Unfortunately, the presented study did not achieve its goal of assessing the impact of PRP application into the wound on the wound’s dehiscence, because there was no case of this complication in either group. However, significant differences between the groups in the scar quality assessment were detected in both patient and doctor POSAS on days 8, 30 and 90 after surgery. In the PRP group, the POSAS scores provided by both the patient and clinician were significantly better (lower) than in the placebo group. These results are presented in detail in Table 3 and Table 4. Additionally, there were observed changes in the POSAS features over time, which showed beneficial effects in the PRP group (Figure 2). Evaluation of the SF-12 questionnaire did not reveal any differences between the groups; therefore, the quality of life was similar in both groups on every recorded day after surgery.

There was no difference in the pain intensity assessment on the VAS recorded after surgery, but PRP patients required fewer paracetamol doses per day than the control group: 11 (47.8%) patients in the PRP group and 3 (13%) patients in the control group required one dose of paracetamol per day, 12 (52.2%) patients in the PRP group and 15 (65.2%) patients in the control group required two doses of paracetamol per day, and none of the patients in PRP group and 5 (21.7%) patients in the control group required three doses of paracetamol per day (*p* = 0.006). There was no difference between the groups in the use of morphine. Only one (4.3%) patient in the PRP group and six (26.1%) patients in the control group required additional use of ketoprofen after CS; however, this difference was not statistically significant (*p* = 0.096). The use of analgetics, together with the VAS scores, is summarized in Table 5.

## 4. Discussion

The present study was aimed at assessing the usefulness of PRP application during CS in improving wound healing and reducing postoperative pain intensity. We demonstrated that PRP application significantly improved wound healing in both short- and long-term assessments. Although it did not influence postoperative pain intensity, it may reduce the use of analgetics after surgery.

Postoperative surgical site infections and wound complications are the most common and costly complications following cesarean delivery, affecting approximately 3 to 15% of women [20,28]. Multiple risk factors for these complications have been identified, including three groups: patient-related, pregnancy-related and surgical risk factors. Obesity, hypertensive disorders of pregnancy, smoking and previous CS are the most common, but also partially modifiable, risk factors [20,29,30]. Among them, obesity is particularly important because of its rapidly increasing incidence worldwide. It is estimated that obesity affects about 30% of women of reproductive age and approximately 13% of pregnant women [31,32]. Smid et al. demonstrated that extremely obese women have increased risk for endometritis, wound infection, wound dehiscence and wound infection-related readmission in comparison to nonobese women [30]. Pregnancy-related risk factors for wound complications after CS include emergency CS, rupture of membranes and chorioamnionitis [20,29]. Surgical risk factors are operating time, surgeon experience, type of suture material, type of incision and abdominal wall closure and the use of antibiotics [14,21]. On the other hand, several evidence-based interventions have been shown to reduce the risk of post-cesarean wound complications, e.g., the administration of antibiotics within 60 min prior to skin incision, using chlorhexidine–alcohol for skin antisepsis and suture closure of the subcutaneous layer in women for whom its thickness is greater than 2 cm [21,33,34,35]. Temming et al. demonstrated that the risk of wound complications was significantly lower in patients who received all the evidence-based interventions during CS in comparison to those who did not (20.3% vs. 28.1%, aRR 0.75, 95% CI 0.58–0.95), but the risk was still high and the authors concluded that there is a need for finding additional interventions that could be able to further reduce this risk [20]. PRP application during CS may be considered as such an additional procedure.

There is a vast body of evidence for the role of PRP in improving wound healing in both animal models and clinical trials [36,37,38,39,40]. PRP contains a high concentration of growth factors and cytokines, such as transforming growth factor-beta (TGF-β), fibroblast growth factor (FGF), platelet-derived growth factor (PDGF), insulin-like growth factors 1 and 2 (IGF-1 and IGF-2), vascular endothelial growth factor (VEGF) and epidermal growth factor (EGF) [3]. The mechanism of PRP action in wound healing is based on stimulating the synthesis of matrix metalloproteinases (MMPs), increasing cutaneous fibroblast growth as well as the production of extracellular matrix (ECM) components including type I collagen and elastin [3]. To the best of our knowledge, there are only two studies evaluating the use of PRP application during CS available to date. Tehranian et al. demonstrated that PRP application during cesarean delivery significantly improved wound healing, as assessed with the edema, ecchymosis, discharge, approximation (REEDA) scale. Additionally, patients treated with PRP experienced a 93% reduction in the VAS score at the end of follow-up (8 weeks after CS), whereas the control group observed only a 79% reduction (*p* < 0.001) [22]. In our study, wound healing was also improved in the PRP group in both short- and long-term assessments. We did not identify any cases of wound dehiscence in our study, which may be due to the small number of patients, but this may also be a good starting point for further research.

Another possible effect of PRP use during CS is postoperative pain reduction. Among various surgical procedures in gynecology together with orthopedics, abdominal surgery and cardiothoracic surgery are among the procedures rated worst by patients in terms of postoperative pain, and CS is placed as the ninth among the most painful surgical procedures [41]. Extensive studies have demonstrated that despite present-day improvements in pain treatment, many patients still suffer from moderate to severe postoperative pain. This is associated with decreased patient satisfaction, delayed postoperative ambulation, the development of chronic postoperative pain and increased incidence of pulmonary and cardiac complications [41,42,43,44]. This is particularly important for women giving birth because of the need to care for a newborn child. Childbirth, independently of other factors, is a risk condition for post-traumatic stress disorder (PTSD), and inappropriate pain treatment after delivery may increase this risk [45]. In both above-mentioned studies on the use of PRP during CS, in contrast to our research, the PRP application significantly reduced postoperative pain [20,23]. These studies, however, did not evaluate the use of analgetics, although it is a good and objective indicator of postoperative pain. The VAS score is based on the patient’s subjective evaluation and may differ between patients because of their earlier life and pain experiences. In our study, women treated with PRP required significantly less use of analgetics, although VAS scores were similar in both groups. Noteworthily, we evaluated VAS scores only in the first 12 h after surgery, whereas other studies continued this evaluation for much longer (weeks or months after delivery). The exact mechanism underlying the antinociceptive action of PRP is not well understood; however, it is quite well understood in relation to neuropathic pain. It is postulated that factors released by platelets and stem cells within platelet-rich plasma lead directly to the elimination of neuropathic pain by triggering enhanced inflammation, followed by the full cascade of the wound healing process, including the regenerative process, resulting in axon regeneration and target reinnervation. This allows axons to take up target-released factors that eliminate nociceptive neuron hyperexcitability and thereby eliminate pain [46]. Improvement in reducing pain intensity and the use of analgetics among patients seem to be particularly important in the face of the opioid crisis in the United States, and further research is needed to assess the potential implementation of PRP into standard procedures aimed not only at improving wound healing but also at reducing postoperative pain and the use of analgetics.

The main limitations of this study are the small number of cases and the fact that there were no cases of wound dehiscence in the study population; thus, the study is underpowered in these aspects. The single-center study design, as well as postoperative pain examination during resting only (and not additionally during coughing), may also be recognized as limitations. The strength of this study is the very homogenous group of patients included. It was a low-risk population—young women, with uncomplicated pregnancies, undergoing elective CS. Another advantage is the use of the POSAS, which is evaluated by both the patient and the clinician, which makes the scar assessment more objective. Moreover, the possible impact of PRP on pain in our study was examined based not only on the VAS, but also on the use of analgetics after surgery. This approach makes pain assessment more reliable in comparison to using the VAS alone, as was performed in the above-mentioned research in the field.

We believe that the results of our study are a good starting point for future research, particularly for evaluation of the potential use of PRP during CS in high-risk patients, such as obese women, those undergoing urgent CS or those with pregnancies complicated by diabetes and hypertension. Confirmation of PRP’s usefulness in preventing wound complications in women undergoing CS may further lead to the implementation of PRP into ERAS protocols and everyday clinical practice, as well as elaborating on new therapeutic and preventive strategies for certain groups of high-risk patients.

## 5. Conclusions

This study demonstrated that PRP application during CS significantly improved wound healing in both short- and long-term assessments. Although it did not influence postoperative pain intensity, it may reduce the use of analgetics after surgery. Further studies are needed to assess the potential benefits of PRP application during cesarean delivery in high-risk patients.

## Figures and Tables

**Table 1:** The demographic and clinical characteristics of the study participants.

	PRP Group *n* = 23	Placebo Group *n* = 23	*p*-Value
Age (years)	31.1 ± 5.2	31.1 ± 5.2	0.700
BMI (kg/m^2^)	26.6 ± 2.9	28.1 ± 3.4	0.191
Nullipara	18 (78.3%)	20 (87.0%)	0.699
Multipara	5 (21.7%)	3 (13.0%)	
Hypothyroidism	10 (43.5%)	9 (39.1%)	0.765
GBS colonization	10 (43.5%)	3 (13.0%)	0.022

BMI—body mass index; GBS—group B streptococcus.

**Table 2:** Delivery and neonatal outcomes of the study participants.

	PRP Group *n* = 23	Placebo Group *n* = 23	*p*-Value
Indications for cesarean section:			0.957
Breech presentation	5 (21.7%)	4 (17.4%)	
Psychiatric (tocophobia)	6 (26.1%)	5 (21.7%)	
Long infertility treatment	4 (17.4%)	3 (13%)	
Fetal macrosomia	1 (4.3%)	1 (4.3%)	
Previous CS because of lack of delivery progress	2 (8.7%)	2 (8.7%)	
Neurologic	1 (4.3%)	4 (17.4%)	
Ophthalmologic	2 (8.7%)	2 (8.7%)	
Orthopedic	1 (4.3%)	0	
Cardiologic	1 (4.3%)	2 (8.7%)	
Gestational age at delivery (weeks)	38.9 ± 0.29	39.0 ± 0.48	0.323
Neonatal birth weight (g)	3460 ± 391	3474 ± 343	0.930
Blood loss:			0.609
300 mL	3 (13%)	4 (17.4%)	
350 mL	17 (73.9%)	13 (56.5%)	
400 mL	3 (13%)	5 (21.7%)	
450 mL	0	1 (4.3%)	
Duration of patient’s hospitalization (days)	5.61 ± 1.31	5.35 ± 1.27	0.299

**Table 3:** Results of scar quality assessment (POSAS) by patient in study groups (presented as mean ± SD).

Parameter	PRP Group *n* = 23	Placebo Group *n* = 23	*p*-Value
Day 8			
Total amount	16.96 ± 1.52	17.57 ± 1.85	0.230
Vascularity	3.57 ± 0.84	3.65 ± 0.94	0.732
Pigmentation	3.00 ± 0.6	2.83 ± 0.83	0.446
Thickness	2.61 ± 0.66	2.91 ± 0.6	0.099
Relief	2.04 ± 0.64	1.96 ± 0.64	0.610
Pliability	3.17 ± 0.58	3.22 ± 0.85	0.842
Surface area	2.57 ± 0.59	3.00 ± 0.6	0.015
Day 30			
Total amount	17.00 ± 1.76	18.09 ± 2.00	0.033
Vascularity	3.57 ± 0.84	3.65 ± 0.94	0.732
Pigmentation	3.22 ± 0.6	3.04 ± 0.83	0.446
Thickness	2.74 ± 0.62	3.13 + 0.69	0.035
Relief	1.96 ± 0.37	2.26 ± 0.54	0.076
Pliability	3.17 ± 0.65	3.26 ± 0.81	0.691
Surface area	2.35 ± 0.71	2.74 ± 0.69	0.028
Day 90			
Total amount	14.91 ± 1.54	16.09 ± 1.68	0.021
Vascularity	2.65 ± 0.65	2.78 ± 0.85	0.607
Pigmentation	2.48 ± 0.59	2.48 ± 0.90	0.999
Thickness	2.57 ± 0.73	3.00 ± 0.52	0.019
Relief	2.13 ± 0.55	2.30 ± 0.56	0.309
Pliability	2.74 ± 0.75	2.74 ± 0.69	0.999
Surface area	2.35 ± 0.49	2.78 ± 0.52	0.015

**Table 4:** Results of scar quality assessment (POSAS) by clinician in study groups (presented as mean ± SD).

Parameter	PRP Group *n* = 23	Placebo Group *n* = 23	*p*-Value
Day 8			
Total amount	15.61 ± 1.08	16.17 ± 1.44	0.180
Vascularity	3.17 ± 0.58	3.13 ± 0.63	0.820
Pigmentation	2.52 ± 0.59	2.65 ± 0.57	0.491
Thickness	3.00 ± 0.60	3.17 ± 0.49	0.333
Relief	1.65 ± 0.49	1.65 ± 0.57	0.999
Pliability	2.65 ± 0.65	2.83 ± 0.72	0.340
Surface area	2.61 ± 0.72	2.74 ± 0.54	0.437
Day 30			
Total amount	15.91 ± 1.24	17.13 ± 1.58	0.004
Vascularity	3.09 ± 0.60	3.13 ± 0.63	0.820
Pigmentation	2.87 ± 0.69	3.13 ± 0.69	0.169
Thickness	3.00 ± 0.60	3.39 + 0.72	0.030
Relief	1.83 ± 0.49	1.78 ± 0.60	0.806
Pliability	2.65 ± 0.65	2.83 ± 0.72	0.340
Surface area	2.48 ± 0.67	2.87 ± 0.63	0.021
Day 90			
Total amount	13.39 ± 1.53	14.74 ± 2.11	0.002
Vascularity	2.48 ± 0.59	2.87 ± 0.69	0.042
Pigmentation	1.87 ± 0.69	1.96 ± 0.56	0.646
Thickness	2.65 ± 0.71	3.04 ± 0.64	0.030
Relief	1.83 ± 0.58	2.00 ± 0.91	0.327
Pliability	2.30 ± 0.56	2.39 ± 0.50	0.633
Surface area	2.26 ± 0.45	2.48 ± 0.51	0.196

**Table 5:** The use of analgetics and the VAS scores in the study population.

	PRP Group *n* = 23	Placebo Group *n* = 23	*p*-Value
Morphine:			
Mean number of doses	9.57 ± 2.86	10.09 ± 1.95	0.738
Total dose number	30.87 ± 9.2	35.43 ± 10.5	0.132
Paracetamol:			0.006
1 dose per day	11 (47.8%)	3 (13%)	
2 doses per day	12 (52.2%)	15 (65.2%)	
3 doses per day	0	5 (21.7%)	
The additional use of ketoprofen	0	6 (26.1%)	0.096
VAS score (mean ± SD):			
Immediately after surgery	3.3 ± 2.23	3.96 ± 2.08	0.299
6 h after surgery	4.96 ± 1.49	5.3 ± 1.52	0.465
12 h after surgery	4.96 ± 1.36	5.13 ± 1.36	0.657

## Data Availability

The data presented in this study are available on request from the corresponding author.

## Supplementary Materials

The following supporting information can be downloaded at: https://www.mdpi.com/article/10.3390/medicina60040628/s1, The original VAS and POSAS forms used during this study.
