# Supplementary material for: The Impact of Platelet-Rich Plasma Application during Cesarean Section on Wound Healing and Postoperative Pain: A Single-Blind Placebo-Controlled Intervention Study
Source: Medicina (Kaunas). 2024 Apr 13;60(4):628. doi: 10.3390/medicina60040628 (PMC11052196; doi:10.3390/medicina60040628)
Supplement: Supplementary file 1 [file medicina-60-00628-s001.zip › medicina-2933819-supplementary.pdf]

Skala oceny bólu pooperacyjnego VAS- Visual Analog Scale

Szanowna Pani,

W związku z udziałem w badaniu naukowym prosimy o odpowiedź na następujące pytania.

1. Jak by Pani oceniła natężenie bólu tuż po zabiegu za pomocą poniższej skali?

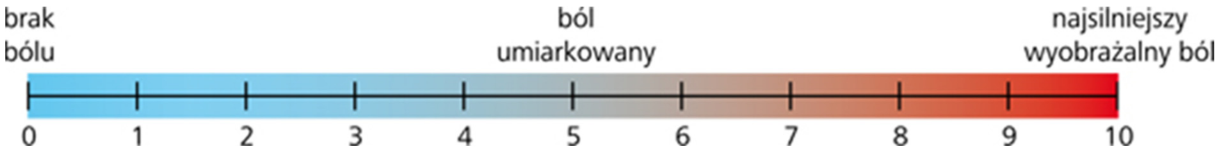

2. Jak by Pani oceniła natężenie bólu 6 godzin po zabiegu za pomocą poniższej skali?

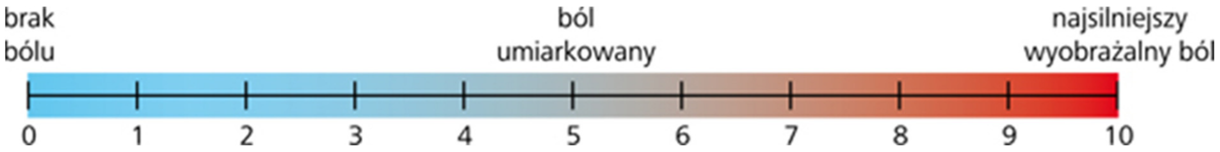

3. Jak by Pani oceniła natężenie bólu 12 godzin po zabiegu za pomocą poniższej skali?

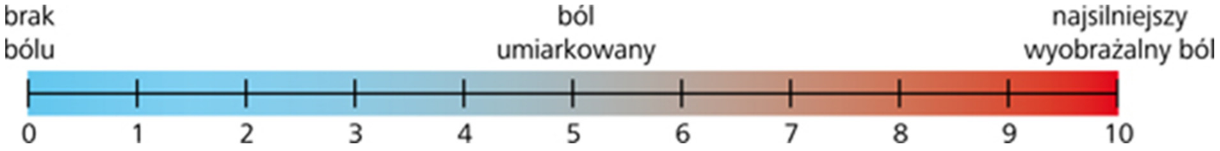

Skala obserwacyjna wyglądu blizn - POSAS The Patient and Observer Scar Assessment Scale

DATA ____

MIEJSCE____

DANE OSOBY OCENIAJĄCEJ__________________________

IMIĘ, NAZWISKO PACJENTA_________________________

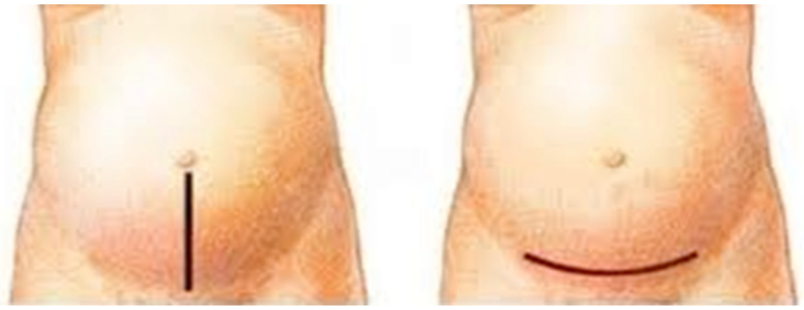

1 = prawidłowy wygląd blizny **10 = najgorzej wyglądająca blizna**

Skala oceny blizny	1	2	3	4	5	6	7	8	9	10
ukrwienie	○	○	○	○	○	○	○	○	○	○
pigmentacja	○	○	○	○	○	○	○	○	○	○
grubość	○	○	○	○	○	○	○	○	○	○
wypukłość	○	○	○	○	○	○	○	○	○	○
giętkość	○	○	○	○	○	○	○	○	○	○
powierzchnia	○	○	○	○	○	○	○	○	○	○

Ogólna ocena blizny	○	○	○	○	○	○	○	○	○	○

WYJAŚNIENIE:

- Skala obserwacyjna POSAS składa się z sześciu elementów: unaczynienia, pigmentacji, grubości, wypukłość, giętkości oraz oceny ogólnej powierzchni.
- Wszystkie elementy są punktowane w skali w zakresie od 1 (jak skóry normalnej) do 10 (najgorszej blizny jaką można sobie wyobrazić).
- Suma sześciu elementów prowadzi do całkowitej punktacji w skali POSAS.
- Kategorie pola są dodawane do każdego elementu.
- Wszystkie parametry powinny być oceniane wyżej niż 1 w stosunku do normalnej skóry na porównywalnej lokalizacji anatomicznej.

• **Obecność ukrwienie naczyń** w tkance blizny ocenianych przez ilość zaczerwienienia badanego przez ilość krwi po powrocie przy ucisku.

• **Pigmentacja**- zabarwienie blizny od pigmentu (melaniny); zastosowania ucisku na skórę z umiarkowanym ciśnieniem, aby wyeliminować wpływ ukrwienia na kolor blizny.

• **Grubość blizny** - średnia odległość między granicą podskórnej i powierzchnią naskórka blizny w wymiarze poprzecznym, czyli w szerokości.

- Wypukłość – wybrzuszenie blizny w stosunku do otaczającej prawidłowej skóry - wysokość.

• **Giętkość** -jędrność badanej skóry marszcząc bliznę między kciukiem i palcem wskazującym.

• **Powierzchnia blizny** w stosunku do pierwotnego obszaru rany.
